# Mycobiota composition and changes across pregnancy in patients with gestational diabetes mellitus (GDM)

**DOI:** 10.1038/s41598-022-13438-0

**Published:** 2022-06-02

**Authors:** Ilario Ferrocino, Valentina Ponzo, Marianna Pellegrini, Ilaria Goitre, Matteo Papurello, Irene Franciosa, Chiara D’Eusebio, Ezio Ghigo, Luca Cocolin, Simona Bo

**Affiliations:** 1 Department of Agricultural, Forestry and Food Science, University of Torino, 10095 Grugliasco, Torino, Italy; 2 Department of Medical Sciences, University of Torino, 10126 Torino, Italy

**Keywords:** Microbial ecology, Microbiome, Gestational diabetes, Fungal ecology

## Abstract

The gut mycobiota has never been studied either during pregnancy or in patients with gestational diabetes (GDM). This study aimed to analyze the fecal mycobiota of GDM patients during the second (T2) and third (T3) trimester of pregnancy and to compare it with the mycobiota of pregnant normoglycemic women (controls). Forty-one GDM patients and 121 normoglycemic women were studied. GDM mycobiota was composed almost exclusively by the Ascomycota phylum; Basidiomicota accounted for 43% of the relative frequency of the controls. *Kluyveromyces* (*p* < 0.001), *Metschnikowia* (*p* < 0.001), and *Pichia* (*p* < 0.001) showed a significantly higher frequency in GDM patients, while *Saccharomyces* (*p* = 0.019)*,* were more prevalent in controls. From T2 to T3, a reduction in fungal alpha diversity was found in GDM patients, with an increase of the relative frequency of *Candida*, and the reduction of some pro-inflammatory taxa. Many associations between fungi and foods and nutrients were detected. Finally, several fungi and bacteria showed competition or co-occurrence. Patients with GDM showed a predominance of fungal taxa with potential inflammatory effects when compared to normoglycemic pregnant women, with a marked shift in their mycobiota during pregnancy, and complex bacteria-fungi interactions.

## Introduction

The human mycobiota is a neglected component of the microbiome, including all the different fungal species living in the human host^1–3^. More than 300 different fungal species are living in the human digestive tract with predominance of the Ascomycota, Basidiomycota and Zygomycota phyla^4^ and, among the genera, *Candida*, *Paecilomyces*, *Penicillum*, *Aspergillus*, *Trichosporon*, *Rhodotorula*, *Cladosporium*, *Aureobasidium, Saccharomycetales*, *Fusarium* and *Cryptococcus*^1,5,6^. Overall, there is a low fecal fungal diversity with a high degree of within- and between-subjects variability^7^. Most species derive from the oral cavity or from diet, being natural food contaminants, and dietary habits, age, gender, and drugs influence the mycobiota composition^6,8^. A low fungal abundance has been suggested to characterize the gut of healthy individuals^9^, but, on the other hand, higher gut fungal abundance has been reported to favorably influence childhood growth^10^, and the gut mycobiota may play beneficial effects for the human host, such as nutrient extraction, vitamin production, modulation of metabolism, and immunoregulation and defense against pathogens^3,11,12^. Conversely, an unbalance in the gut fungal composition (“fungal dysbiosis”) and colonization by fungal species has been reported in specific diseases, above all in immunocompromised patients^1,5^. Recently, a growing number of papers is reporting associations among specific gut fungal genera and chronic diseases, such as Alzheimer’s disease^13^, irritable bowel syndrome (IBS)^14^, inflammatory bowel diseases (IBD)^2,4,15–17^, alcoholic liver diseases^18^, colorectal adenoma and cancer^19,20^, the progression of pancreatic cancer ^21^, mold-induced asthma ^22^, bronchiectasis ^23^, carotid atherosclerosis ^24^, diabetes mellitus ^25–27^, and even weight loss ^28^ as well as many other post-surgical, nosocomial conditions or immunocompromise-induced diseases ^3,4^.

It is well known that the human microbiome changes in physiological conditions, such as pregnancy, and might play a role in gestational dysmetabolic conditions as well, for example, gestational diabetes mellitus (GDM)^29^. Indeed, the potential role of the gut mycobiota in GDM remains largely unexplored. Similarly, very few and contrasting data are available about the associations between dysmetabolic conditions and the mycobiota. Relationships have been reported between *Candida* and diabetes mellitus^25,26^, but not between *Candida* and obesity^28^.

The aims of the present observational study were, therefore, (i) analyzing the fecal mycobiota of patients with GDM during the second and the third trimester of pregnancy and (ii) comparing it with the mycobiota of pregnant normoglycemic women.

## Results

Out of 50 GDM patients, 9 women were lost at follow-up or did not return the stool sample. Out of 150 normoglycemic women, 29 women did not return the stool sample or collected and/or stored the stool sample incorrectly. The baseline clinical characteristics of the participants did not differ from those of the women who were lost (data not shown). Therefore, 41 GDM patients and 121 normoglycemic women were analyzed. Their clinical characteristics are reported in Supplementary Table S1. Most GDM patients were overweight women, with excessive fat intake (> 40% total daily energy instead of the recommended < 35%) and low fiber consumption (15 g/day, i.e., ~ 9 g/1000 kcal instead of the recommended 12.6–16.7 g/1000 kcal). From enrolment (T2) to the study end (T3), weight and Body Mass Index (BMI) increased, and metabolic and inflammatory patterns of participants worsened, as usually occurs during the third trimester of pregnancy (CRP from 4.1 to 4.5 mg/L, glycated hemoglobin from 4.6 to 4.9%, total cholesterol from 234 to 257 mg/dL, triglycerides from 173 to 259 mg/dL, Supplementary Table S1). Normoglycemic women were significantly younger and showed, as expected, lower weights, BMI, and fasting glucose values.

### Mycobiota composition in GDM patients and in normoglycemic women during the second trimester of pregnancy

A total of 14.007.462 paired reads were obtained by sequencing. After quality filtering, a total of 13.356.188 reads were used, with an average value of 61.266 reads/sample, and a mean sequence length of 465 bp. Alpha diversity index showed a satisfactory coverage for all samples with an average value of 99%. Alpha rarefaction curves reached a plateau, indicating that most biodiversity was captured by the applied analysis (Supplementary Fig. 1).

**Figure 1:**
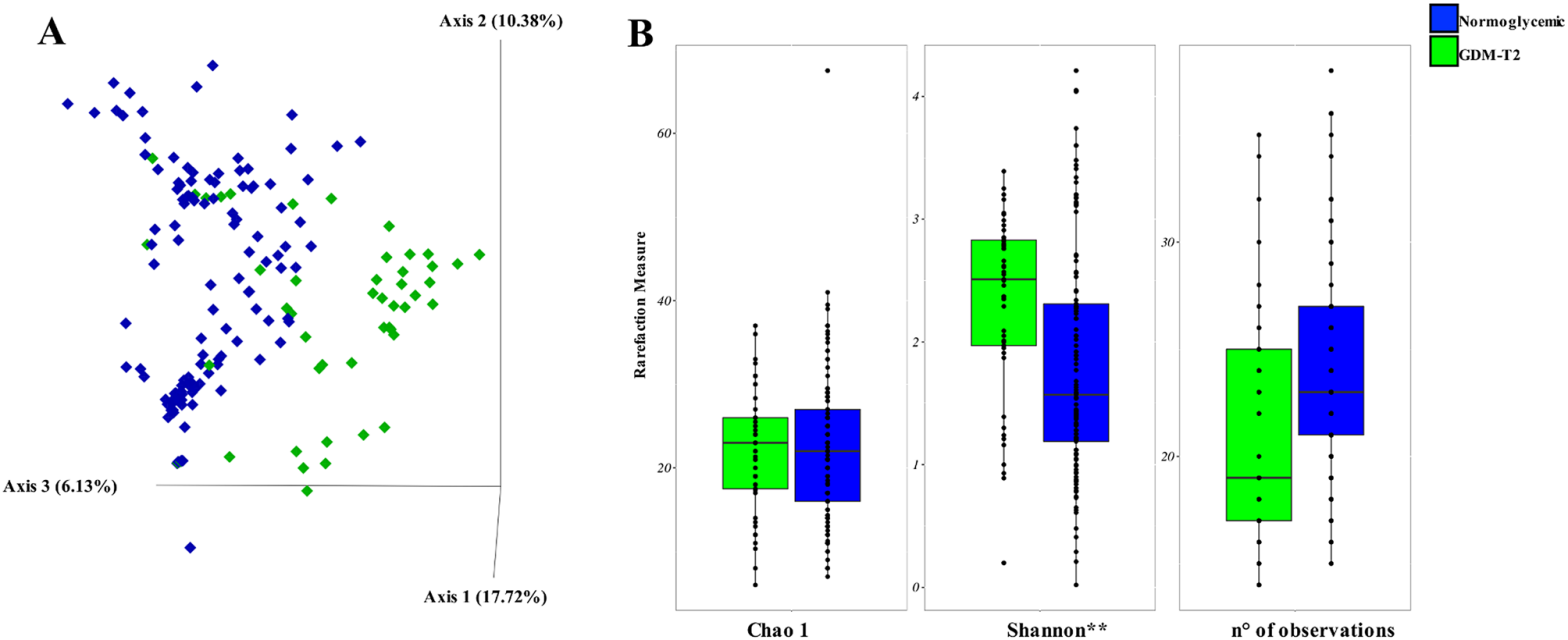
Mycobiota diversity during pregnancy in normoglycemic and GDM women during the second trimester of pregnancy. (**A**) Principal coordinate analysis (PCoA) of Bray–Curtis distance with each sample represented as a diamond and color code according to status (normoglycemic or GDM). (**B**) Alpha diversity measure of the mycobiota in normoglycemic or GDM participants. Between-group differences were assessed by the Mann–Whitney test: **p < 0.01.

Beta and alpha diversity analyses in GDM and normoglycemic women are shown in Fig. 1. Bray–Curtis distance matrix showed a significant separation between GDM and controls (Fig. 1A, pairwise PERMANOVA *p* = 0.001). Alpha-diversity by the Shannon index (Fig. 1B) was significantly higher in GDM patients than controls (*p* < 0.001).

**Figure 2:**
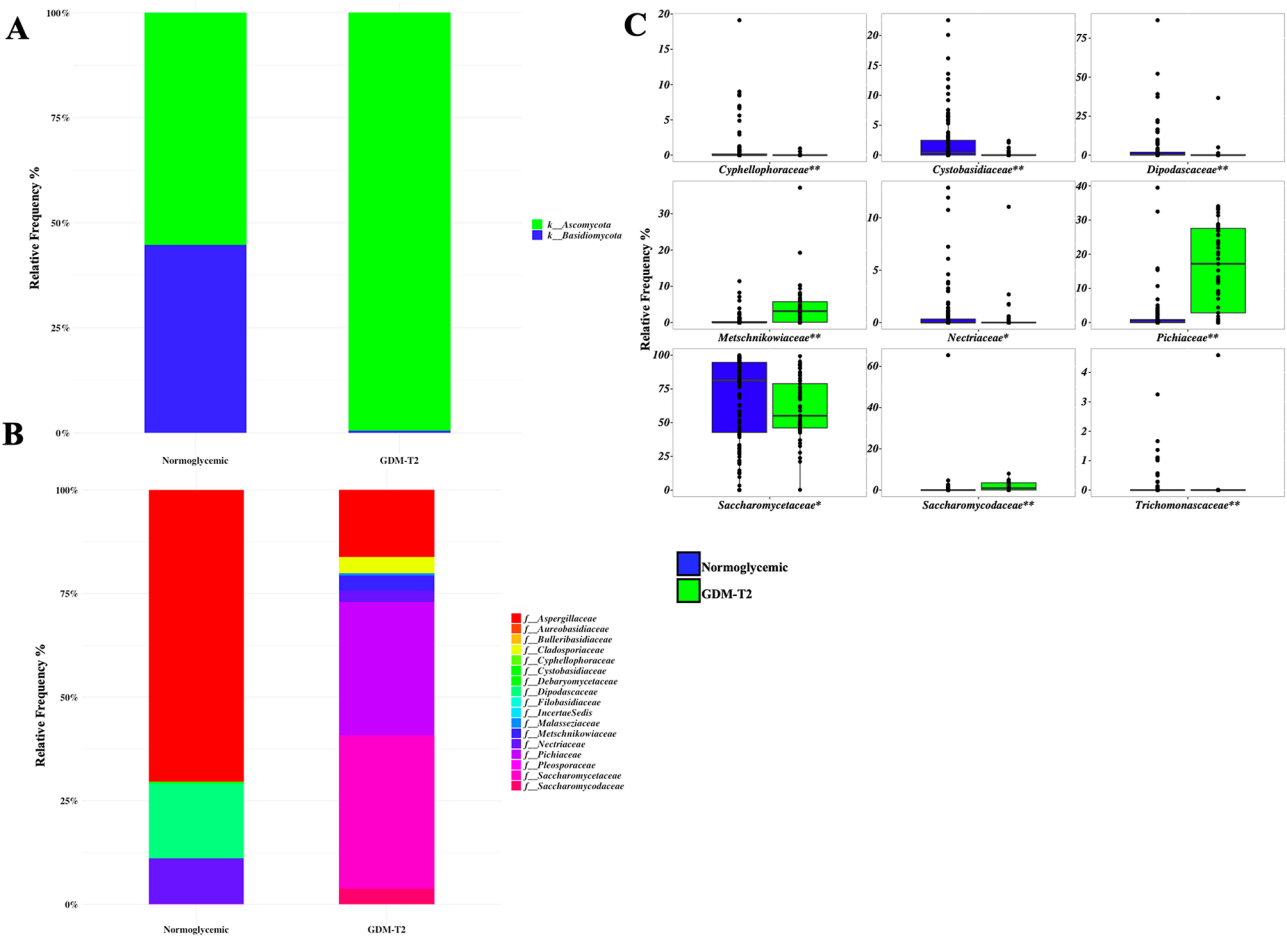
Plot A and Plot B: Global composition of mycobiota at phylum (**A**) and family levels (**B**) during the second trimester of pregnancy in normoglycemic and GDM women. Only ASVs with with a relative frequency > 1% in at least 10% of subjects are shown. Normoglycemic or GDM participants are labelled on the x-axis and expressed as the relative Amplicon Sequence Variants (ASVs) frequency. Plot C: boxplots of the ASVs at family level which were significantly different between normoglycemic and GDM women (Mann–Whitney test, *p < 0.05; **p < 0.01).

**Figure 3:**
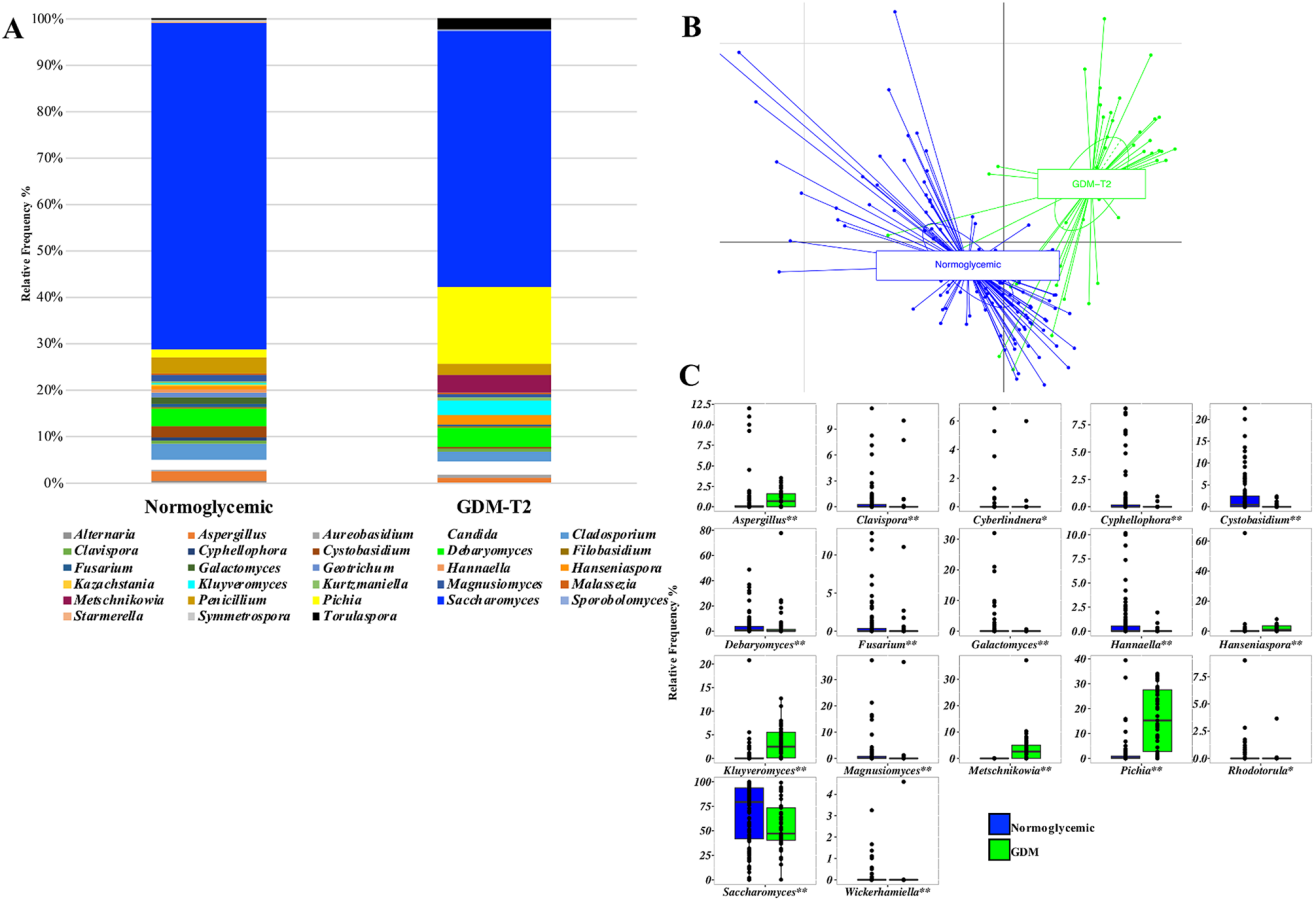
(**A**) global composition of mycobiota at genus level during the second trimester of pregnancy. Only ASVs with a relative frequency > 1% in at least 10 samples are shown. Normoglycemic or GDM subjects are labelled on the x-axis and expressed as the relative Amplicon Sequence Variants (ASVs) frequency. (**B**) Principal Component Analysis (PCA) based on ASVs relative abundance at genus level of normoglycemic and GDM women. (**C**): boxplots of the ASVs at genus level which were significantly different between normoglycemic and GDM women (Mann–Whitney test, *p < 0.05; **p < 0.01).

The mycobiota of patients with GDM was composed almost exclusively by the Ascomycota phylum (Fig. 2A); Basidiomicota accounted for 43% of the relative frequency of the mycobiota of normoglycemic women. *Saccharomycodaceae*, *Pichiaceae*, *Debaryomycetaceae*, *Aspergillaceae*, *Dipodascaceae, Cladosporiaceae* and *Metschnikowiaceae* were the most prevalent families (Fig. 2B). The relative frequency of *Pichiaceae* and *Metschnikowiaceae* was significantly higher, while that of *Saccharomycetaceae* and several minor ASVs were lower in GDM patients than in normoglycemic women; the between-group significant differences at family level are described in Fig. 2C. The predominant genera were: *Saccharomyces* (54% and 68% of the relative frequency in GDM and normoglycemic women, respectively), *Pichia* (16% and 2%), *Debaryomyces* (4.2% and 3.8%)*, Metschnikowia* (3.6% and 0%)*, Penicillium* (2.3% and 3.0%), *Cladosporium* (2.3% and 3.2%), *Geotrichum* (0.1% and 0.9%), *Candida* (2.5% and 1.96%), *Kluyveromyces* (3.1% and 0.4%), *Torulaspora* (2.2% and 0.3%)*,* and *Aspergillus* (0.9% and 1.7%) (Fig. 3A, Supplementary Figure S2 and Supplementary Table 2). At genus level, the Principal Component Analysis (PCA) showed a clear separation between GDM and normoglycemic women (Fig. 3B, *p* = 0.005). *Aspergillus*, *Hanseniaspora*, *Kluyveromyces*, *Metschnikowia*, and *Pichia* showed a significantly higher frequency in GDM patients, while *Saccharomyces, Clavispora, Cystobasidium, Debaryomyces, Fusarium* and other minor ASVs were more prevalent in normoglycemic women (Fig. 3C). In a multiple regression model, adjusted for age and BMI, *Kluyveromyces* (*ß* = 3.18; ES = 0.44, *p* < 0.001), *Metschnikowia* (*ß* = 4.17; ES = 0.56, *p* < 0.001), and *Pichia* (*ß* = 16.8; ES = 1.37, *p* < 0.001) remained directly associated with GDM and the relative frequency of *Saccharomyces* (*ß* = − 12.9; ES = 5.42, *p* = 0.019), *Clavispora* (*ß* = − 0.62; ES = 0.26, *p* = 0.018), and *Cystobasidium* (*ß* = − 2.23; ES = 0.66, *p* < 0.001) inversely associated.

### Machine Learning Procedure for fungal discrimination between normoglycemic women and GDM patients during the second trimester of pregnancy

**Figure 4:**
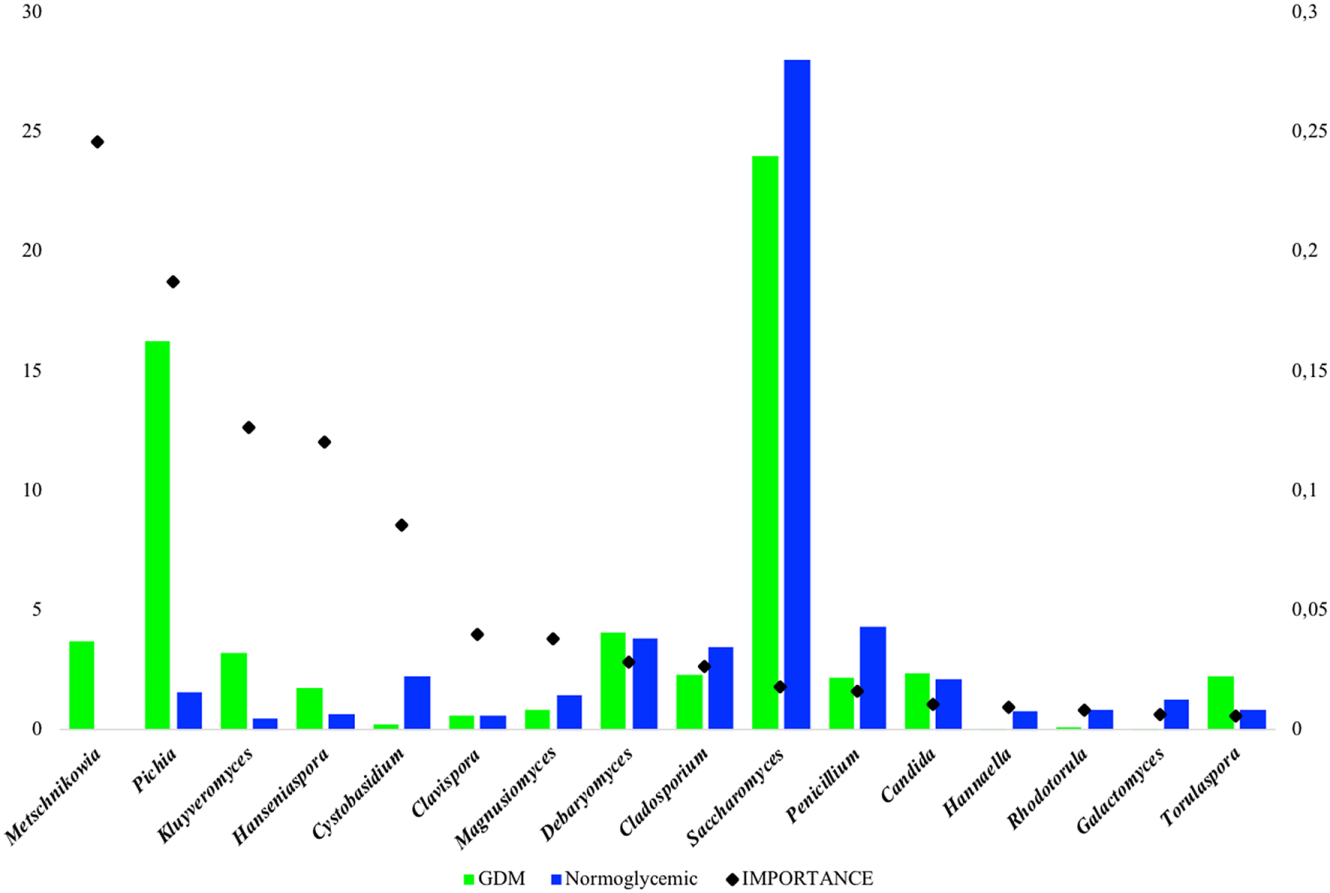
List of the ASVs discriminating GDM from normoglycemic women. For each ASV, the average percentage of frequency is represented by a bar, color-coded according to the groups (left scale). The importance in discriminating of each ASV is represented by a black dot (right diamond).

The random forest model was used to assess the predictive ability of the ASVs at genus level in discriminating between GDM patients and normoglycemic women. The ROC analysis, performed in order to verify the accuracy of the model, showed that gut mycobiota differentiated GDM from normoglycemic women, with an AUC equal to 0.91. The relative frequency and the importance of the first 17 ASVs in discriminating between the two groups are shown in Fig. 4. The importance of the ASVs was not related with their relative frequency. Several ASVs with low frequency showed a high discriminative potential (such as *Metschnikowia, Candida, Pichia, Kluyveromyces, Debaryomyces* and *Torulaspora* for GDM patients, and *Cladosporium, Saccharomyces, Penicillium, Aspergillus* and *Galactomyces* for normoglycemic women).

### Changes in the mycobiota composition in GDM patients from the second to the third trimester of pregnancy

**Figure 5:**
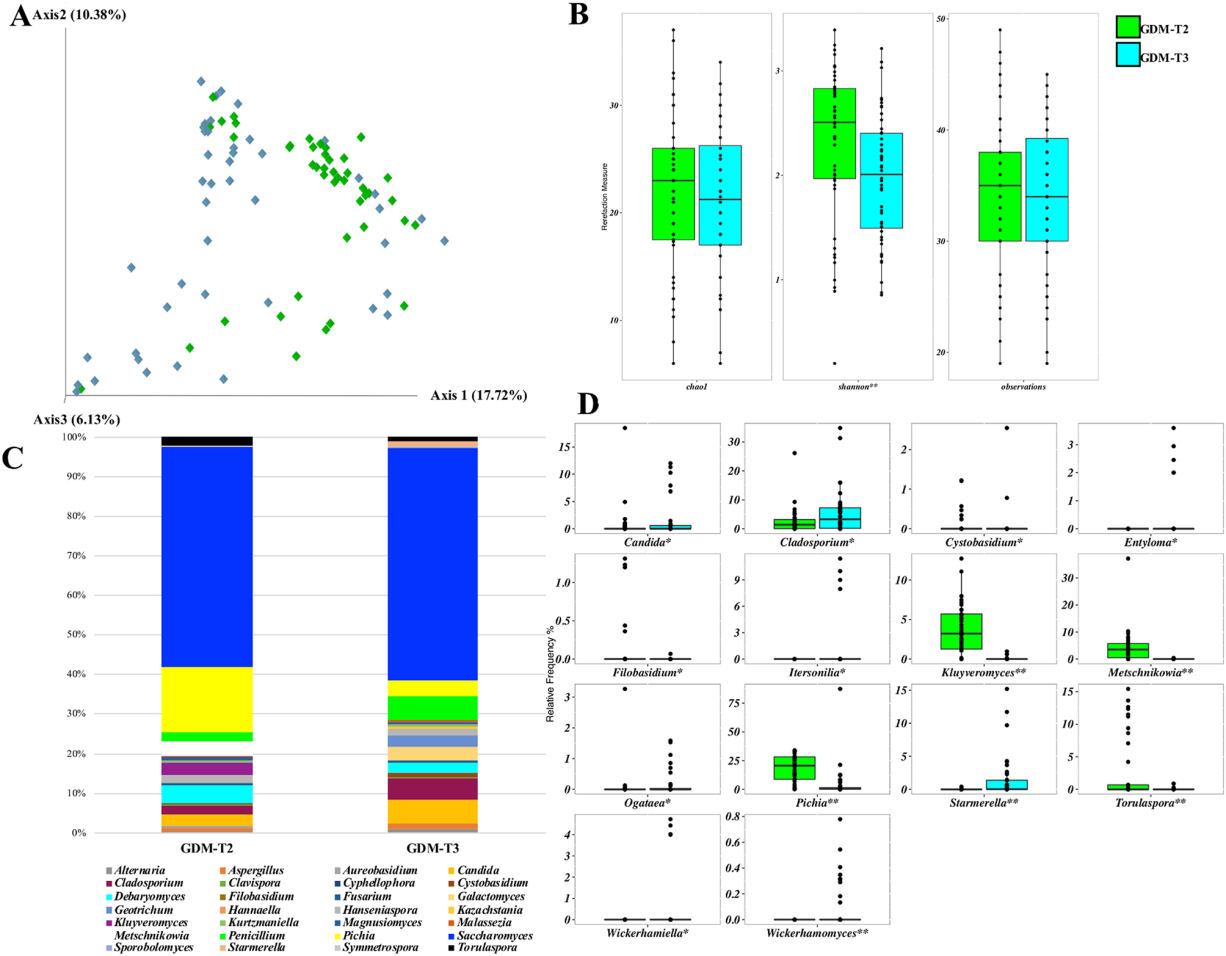
Beta (**A**) and alpha (**B**) diversity measures of the gut mycobiota of GDM patients at the second (T2) and third trimester (T3) of pregnancy. (**C**) global composition of mycobiota at genus level. Only ASVs with a relative frequency > 1% in at least 10 samples are shown. (**D**) boxplots of the ASVs at genus level which were significantly different within GDM women during the second (T2) and third (T3) trimester of pregnancy (Wilcoxon matched pairs test, *p < 0.05; **p < 0.01).

Beta diversity differed between the second and the third trimester of pregnancy (Fig. 5A, PERMANOVA *p* = 0.001), and a reduction in alpha diversity measure by Shannon index was found during pregnancy (Fig. 5B, *p* = 0.003). The relative frequency of genera in the second and third trimesters was respectively *Saccharomyces* (53% and 57%), *Pichia* (18% and 4%), *Metschnikowia* (4% and 0.01%), *Kluyveromyces* (3% and 0.01%), *Debaryomyces* (2% and 3%), *Cladosporium* (2% and 6%), *Penicillium* (2% and 3%), *Torulaspora* (2% and 0.01%), *Candida* (1% and 4%), *Geotrichum* (0.01% and 3%), and *Galactomyces* (0.01% and 4%) (Fig. 5C and Supplementary Table S2). During the third trimester of pregnancy, a significant increase in the relative frequency of *Candida*, *Cladosporium*, *Starmerella*, *Wickerhamiella* and *Wickerhamomyces* was detected (Fig. 5D)*.* On the other hand, the relative frequency of *Cystobasidium*, *Filobasidium*, *Kluyveromyces*, *Metschnikowia*, *Pichia* and *Torulaspora* was reduced with respect to the second trimester of pregnancy.

### Associations between mycobiota composition and dietary and clinical variables in GDM patients in the second trimester of pregnancy

**Figure 6:**
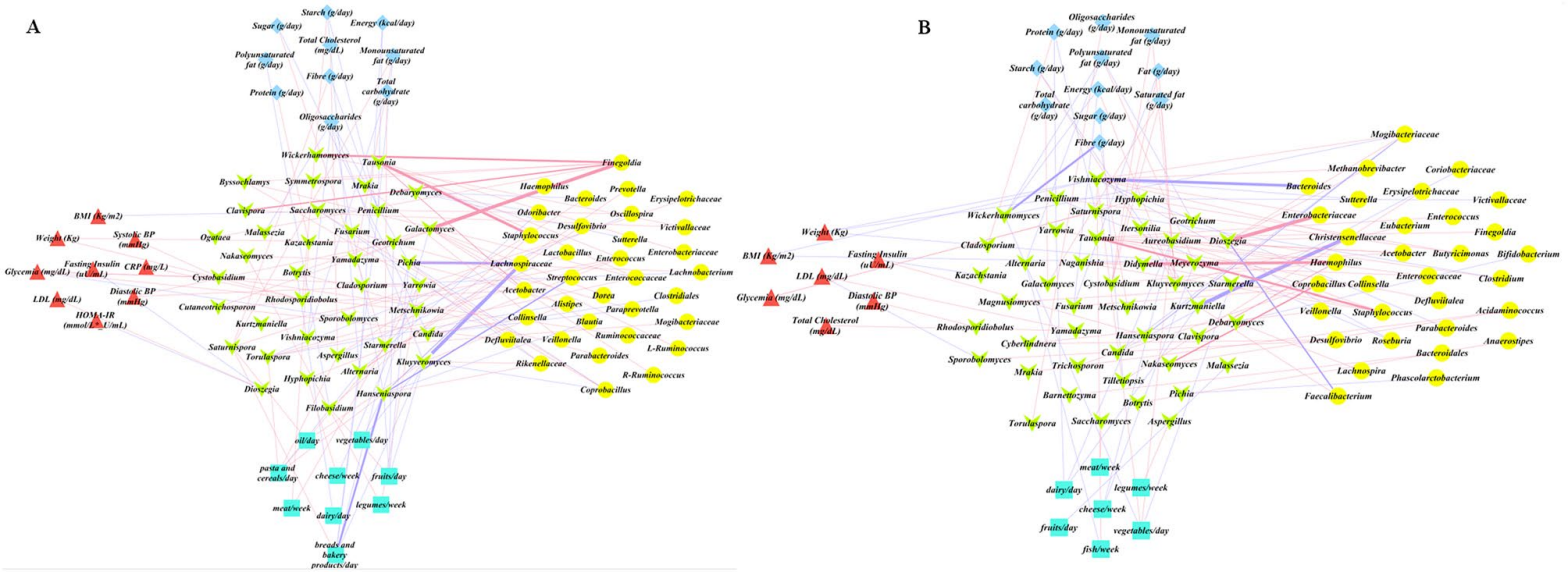
Correlation between mycobiota (green arrow), microbiota (yellow circle), dietary intake (light blue diamond), food frequency (gray square) and clinical variables (red triangle) in GDM patients in the second (**A**) or third trimester (**B**) of pregnancy. Correlation network shows the significant relationships based on Spearman’s correlations. Colors of the edges indicate positive (blue) or negative (red) correlations.

In GDM patients, *Starmerella* was associated with intakes of total carbohydrates (Rho = 0.37; *p* = 0.01) and sugars (Rho = 0.53; *p* < 0.001) (Fig. 6A). *Penicillium* showed an inverse relationship with dietary fiber (Rho = − 0.45; *p* = 0.002), monounsaturated fats (Rho = − 0.43; *p* = 0.004) and total energy intake (Rho = − 0.40; *p* = 0.009). The weekly frequency of meat consumption was directly correlated with *Kazachstania* (Rho = 0.38; *p* = 0.01) and *Yarrowia* (Rho = 0.40; *p* = 0.008), while *Hanseniaspora* was inversely associated with the daily consumption of bread (Rho = − 0.53; *p* < 0.0001), and vegetables (Rho = − 0.46; p = 0.002) and directly with daily consumption of pasta (Rho = 0.30; *p* = 0.049). *Metschnikowia*, *Torulaspora* and *Aspergillus* were related to the daily consumption of pasta and cereals (Rho = 0.31; *p* = 0.046; Rho = 0.32; *p* = 0.038; Rho = 0.35; *p* = 0.02, respectively). Furthermore, *Geotrichum* was directly associated with weight (Rho = 0.34; *p* = 0.028), *Yarrowia* and *Geotrichum* with diastolic blood pressure (Rho = 0.31; *p* = 0.045, and Rho = 0.41; *p* = 0.007; respectively), *Yarrowia* and *Metschnikowia* (Rho = 0.33; *p* = 0.031, and Rho = 0.41; *p* = 0.007, respectively) with fasting blood glucose values, *Aspergillus* with serum cholesterol (Rho = 0.38: *p* = 0.01) and *Penicillium* was inversely associated with BMI (Rho = − 0.36; *p* = 0.02). In a multiple regression model, many of these associations remained statistically significant (Table 1).

**Table 1:** Statistically significant associations between mycobiota composition and dietary and clinical variables in patients with GDM.

	Beta	95%CI	*P*
**Second trimester**			
Total energy (kcal/day)			
*Penicillium*	− 0.004	0.0016	0.025
Fiber (g/day)			
*Penicillium*	− 0.32	0.089	< 0.001
Monounsaturated fats (g/day)			
*Penicillium*	− 0.15	0.043	0.001
Meat (times/week)			
*Yarrowia*	0.55	0.019	0.006
Bread (times/day)			
*Hanseniaspora*	− 0.97	0.0238	< 0.001
Pasta and other cereals (times/day)			
*Aspergillus*	0.96	0.34	0.008
Vegetables (times/day)			
*Hanseniaspora*	− 0.57	0.027	< 0.001
Fasting glucose (mg/dL)			
*Metschnikowia*	0.105	0.049	0.040
Serum total cholesterol (mg/dL)			
*Aspergillus*	0.015	0.005	0.008
**Third trimester**			
Monounsaturated fats			
*Kluyveromyces*	0.0063	0.0029	0.036
Fasting insulin (µU/mL)			
*Hanseniaspora*	0.17	0.068	0.017

Multiple regression model evaluating the association between the mycobiota relative frequency (dependent variable) and the dietary and clinical variables (independent variables), after adjustments for age and BMI.

### Associations between mycobiota composition and dietary and clinical variables in GDM patients in the third trimester of pregnancy

The relative frequency of *Kluyveromyces* was directly associated with dietary fats (Rho = 0.42; *p* = 0.005), monounsaturated (Rho = 0.41; *p* = 0.007) and saturated fats (Rho = 0.33; *p* = 0.032), total energy intake (Rho = 0.38; *p* = 0.01), and starch (Rho = 0.32; *p* = 0.040) (Fig. 6B); *Metschnikowia* was associated with fiber (Rho = 0.35; *p* = 0.02), *Aureobasidium* with the daily consumption of vegetables (Rho = 0.44; *p* = 0.003), while *Aspergillus* with the weekly consumption of legumes (Rho = 0.31; *p* = 0.045) (Fig. 6B). Fasting insulin levels were associated with the relative frequency of *Hanseniaspora* (Rho = 0.31; *p* = 0.049), and fasting glucose with *Saturnispora* (Rho = 0.35; *p* = 0.023)*.* In a multiple regression model, the association between *Kluyveromyces* and monounsaturated fats and the association between *Hanseniaspora* and fasting insulin remained statistically significant (Table 1).

### Bacteria–fungi associations in GDM patients

We analyzed the gut bacteria-fungi associations; the gut microbiota was previously characterized^30^. A different pattern of bacteria-fungi associations was observed in GDM patients at T2 (Fig. 6A) and T3 (Fig. 6B). A complex relationship between bacteria and fungi in the correlation network was more evident at T2 than at T3 (Fig. 6A, FDR < 0.02). At T2, *Aspergillus* displayed co-exclusion pattern with *Lactobacillus* while co-occurred with *Bacteroides*. *Debaryomyces* co-excluded *Bacteroides,* while *Galactomyces* co-occurred with *Blautia; Dorea* and *Ruminococcus*. *Kluyveromyces* and *Metschnikowia* co-excluded *Lachnospira*. A significant inverse association between *Hanseniaspora* and *Blautia* and between *Lachnospiraceae* with *Hanseniaspora*, *Kluyveromyces* and *Pichia* was detected while *Finegoldia* co-occurred with *Galactomyces* (Fig. 6A, FDR < 0.02)*.* At T3, the direct associations between *Kluyveromyces* and *Eubacterium*, *Fusarium* and *Collinsella*, *Penicillium* and *Roseburia*, and the inverse relationship between *Penicillium* and *Butyricimonas*; between *Bacteroides* and *Vishniacozyma*; between *Christensenellaceae* and *Kurtzmaniella* were identified (Fig. 6B, FDR < 0.02).

## Discussion

GDM patients showed a different mycobiota composition than normoglycemic women, with increased alpha diversity and predominance of Ascomycota, in particular, *Metschnikowia, Pichia* and *Kluyveromyces*, and reduced relative frequency of *Saccharomyces*. From the second to the third trimester of pregnancy, the mycobiota composition and alpha diversity changed in GDM patients. Many associations between fungi and dietary and clinical variables were detected. Finally, several fungi and bacteria showed inverse or direct relationships, suggesting respectively, competition or co-occurrence.

### Gut mycobiota signature between GDM patients and normoglycemic women

An increase in alpha diversity was observed in GDM patients when compared to normoglycemic women. No data on fungal alpha diversity are available, at present, in pregnant women, and very few studies have assessed this topic. Individuals with overweight or obesity showed an increased fungal count, but a slightly decreased diversity^6,28^, as well as patients with IBD^31^, IBS^14^, *Clostridium difficile* infection^32^, and psychiatric diseases^33,34^. Indeed, these data are contrasting, since the same authors did not outline differences in alpha diversity in samples of patients with Crohn disease^31^ and of IBS-like mice^14^. Furthermore, other authors failed to find differences between patients with ulcerative colitis^35^ or neurodevelopmental conditions^36^ and healthy subjects. A possible explanation for the increased alpha diversity might be the increased relative frequency of the taxa potentially proinflammatory in our GDM patients when compared to normoglycemic controls (see below). Otherwise, the dietary habits of GDM women, which have been reported to differ from those of normoglycemic pregnant women^29,37^, might be implicated. Unfortunately, lack of reliable nutritional data in our normoglycemic women has not allowed us to make such a comparison.

GDM patients were characterized by the predominance of Ascomycota, differently from the normoglycemic women, who displayed ~ 50% relative frequency of Basidiomycota. Accordingly, Ascomycota phylum has been shown both to predominate in individuals with obesity and to be negatively correlated with the Basidiomycota phylum^28^. Several ASVs showed a high discriminative potential between GDM and normoglycemic women, in particular, *Metschnikowia, Pichia* and *Kluyveromyces* in GDM patients. *Metschnikowia* is an environmental, plant-related yeasts previously isolated from human samples (saliva, blood, vagina, and rectum) and related to several skin pathologies^38^. Strains belonging to *Metschnikowia* are able to exert an antagonist effect against microbes and fungi due to the production of pulcherrimin, a pigment closely linked to its antagonistic capacity, the production of which is promoted by the presence of simply sugars, such as glucose, galactose and disaccharides^39^. Therefore, it could be hypothesized that in the presence of increased concentrations of glucose, *Metschnikowia* might exert antagonist effects against other taxa, thus justifying its predominance in GDM towards normoglycemic women. *Pichia*, a member of the oral and gut microbiome^40^, was shown to be increased in *Clostridium difficile* infection when compared to healthy individuals^41^ and has implicated in infant gut mycobial dysbiosis associated with atopic disease^42^. *P. kudriavzevii* belonging to *Pichia* genus was associated with an increased inflammatory response and a reduced abundance of short chain fatty acids (SCFAs) in mice^43^. It was shown that the gut dysbiosis with depletion in SCFA-producing bacteria, which characterizes GDM patients^44^, can increase the predominance of inflammatory fungal taxa, such as *Pichia*, that release an inhibitory molecule with antagonistic effects against the other fungi^40^, thus explaining its increased relative frequency in the GDM patients. *Kluyveromyces* (a common dairy yeast) was a discriminant taxon in our GDM patients. It was reported to be also discriminant in Crohn’s disease^35^. Members from this genus are able to produce volatile sulfur compounds via methionine aminotransferase^45^. Higher levels of sulfuric compounds are considered to play a role in chronic inflammatory diseases, such as IBDs and cancers^46^.

*Aspergillus*, *Hanseniaspora*, *Candida,* and *Torulaspora* showed an increased, though not significant, higher frequency in GDM patients than in normoglycemic women. In particular, no significant difference was found in the relative frequency of *Candida* spp., which have been suggested to increase in diabetes mellitus and inflammatory gut diseases, and to be linked to decrease SCFA levels in the gut^6,25,26,28^. Indeed, other studies, similarly to our findings, did not point out differences in this taxon in the presence of conditions associated with insulin resistance, such as obesity^6,28^.

Normoglycemic women were characterized by an increased relative frequency of *Saccharomyces*, and other minor taxa, such as *Clavispora* and *Cystobasidium*. *Saccharomyces* is a stable yeast in the gut of healthy individuals, even after consumption of *Saccharomyces* free-diet^9,47,48^. Strains belonging to this genus have been used to treat gastrointestinal disorders such as IBD and IBS^49,50^. Patients with IBD and obesity showed highest levels of anti *S. cerevisiae* antibodies and lower abundance of *Saccharomyces* in the gut^31^. Furthermore, *Saccharomyces* coexists with beneficial SCFA-producing bacteria, such as *Faecalibacterium*, *Lachnospiraceae* and *Ruminococcaceae*^51^, thus suggesting a favorable anti-inflammatory role. Finally, physiologically relevant concentrations of SCFAs seem to be fungistatic^52^.

### Gut mycobiota changes during GDM

During the course of pregnancy, we have observed both a reduction in the alpha diversity value and changes in the mycobiota composition. The reduction of alpha diversity within the patients with GDM is in line with the remarkable hormonal, immunological, and metabolic changes taking place during the third trimester to promote maternal weight gain, increasing circulating pro-inflammatory cytokines, and insulin resistance, which are accentuated in the presence of gestational hyperglycemia^29^. Among the changes in the mycobiota composition occurring between the second and the third trimester of pregnancy, the reduction in the relative frequency of *Pichia*, *Metschnikowia*, *Kluyveromyces*, and the increase in *Candida*, *Cladosporium* and *Starmerella* are the most relevant variations we observed. An inverse relationship between *Candida* and *Pichia* has already been reported^53^, with a reduction of the latter coinciding with an increase in *Candida* colonization, and vice versa. Recently, *Candida* spp have been demonstrated to play a key role in induction host-protective antifungal IgG antibodies that are protective against systemic fungal infection^12^. Thus, competitive effects among fungal taxa may be hypothesized.

### Associations between mycobiota, nutrient intakes, and metabolic variables

Our GDM women ate an excessive amount of fat and less than the recommended dietary fiber intake. Most gut fungi derive from foods^4^, in particular, *Penicillium,* and *Aspergillus* were associated with a plant-based diet^54^. On the other hand, an enrichment of *Penicillium* was observed in humans consuming an animal-based diet^55^ and in mice fed with a high-fat diet^56^. Moreover, *Aspergillus* has been linked to adiposity and related metabolic disorders including insulin resistance, arterial blood pressure, and inflammation^28^. Accordingly, in our GDM patients, the relative frequency of *Aspergillus* was associated with the consumption of pasta and cereals, while *Penicillium* was inversely associated with fiber and monounsaturated fat intake and with serum cholesterol levels.

Furthermore, we found a direct relationship between *Yarrowia* and meat consumption; strains belonging to *Yarrowia* (*Y. lipolytica*) are commonly found in poultry products, ham, beef, and sausages^57^ as well as in dairy foods. *Kluyveromyces* is a dairy potential probiotic^58^ whose relative frequency was found to be associated with the intake of monounsaturated fats in the third trimester. *Hanseniaspora* resulted inversely correlated with plant-derived foods and, in the third trimester of pregnancy, with fasting insulin. In line, six-weeks of a high-fat diet induced an increase in this taxon in mice^56^. Finally, *Metschnikowia* (a plant related yeasts) was associated with fasting glucose, since increased concentrations of glucose might favor its growth and the exertion of antagonist effects against other taxa^39^.

### Bacteria–fungi interactions in GDM patients

The chronic inflammatory status associated with gestational hyperglycemia^59^ impacts on the microbiota composition^60^, potentially modifying the interaction between bacteria and fungi, by causing changes in their relative abundances. We have previously found several associations between pro-inflammatory bacteria (*Bacteroides, Collinsella*, and *Sutterella*) and metabolic/inflammatory variables across pregnancy in our GDM patients^30^.

Complex relationships between bacteria and fungi have been reported in humans, whose biological significance is still uncertain, but might be an opportunity to modulate the gut taxa^7,31,48^. Accordingly, we found that fungi likely associated with a pro-inflammatory state, such as *Metschnikowia*, excluded beneficial bacteria such as *Lachnospira* and *Lactobacillus. Aspergillus* co-occurred with *Bacteroides*, probably because members of the genus *Aspergillus* can produce extracellular polysaccharides and, consequently, favor the presence of *Bacteroides*, which are polysaccharide degraders^61^. Furthermore, *Hanseniaspora,* which is associated with the consumption of fruits and sugary foods, was inversely associated with *Lachnospiraceae* family and in particular with *Blautia*, whose relative abundance was related to a higher insulin sensitivity in our GDM patients^30^, and *Debaryomyces*, microorganisms with potential health benefit deriving from dairy and fermented foods^62^ co-exclude *Bacteroides*. *Lachnospiraceae* family co-excludes several yeasts, and an abnormal increase in the former was reported to lead to excessive energy uptake from indigestible polysaccharides^63^.

However, the bacteria-fungi relationships were quite complex, and our GDM patients showed the concomitance of pathogenic fungi and pro-inflammatory bacteria, but also the co-occurrence of beneficial fungi and bacteria. Thus, the opportunistic pathogen *Fusarium* (a plant pathogen from environment^40,64,65^) co-occurred with the pro-inflammatory *Collinsella*^66^ which displays the ability to detoxify trichothecene mycotoxins from *Fusarium*^67^. Similarly, the beneficial *Galactomyces,* deriving from dairy fermented foods^64^, co-occurred with beneficial bacteria, such as *Blautia*. The health-associated taxa, *Christensenellaceae* co-exclude the presence of *Kurtzmaniella* which was reported to be associated with high-fat diet^68,69^.

This area of research is at the beginning, but it is quite intriguing owing its potential implications on health. In particular, the study of known microbiome interactions with human health, comprising pregnancy and preterm birth, inflammatory bowel diseases, and stressors that affect individuals with prediabetes, have been considered to serve as models of ‘typical’ microbiome-associated conditions of broad interest to the research community^70^.

### Clinical implications

The transfer of fungal phenotypes from the mother to the offspring has been reported^47^ and early fungal colonization was hypothesized to impact on the development of diseases later in life^3^. Increased fungal abundance in children was associated with increased height velocity and reduced BMI^10^, and the mycobiome characteristics have been implicated in obesity and many inflammatory and allergic diseases during childhood^71–74^.

The development of strategies that promote a beneficial mycobiota in pregnancy might be an opportunity to modulate the inflammatory status and insulin resistance characterizing gestational hyperglycemia, and the future health of the offspring^11^.

### Limitations and strengths

The limitations of the present study should be acknowledged. Finding one method able of sufficiently extracting DNA from all fungal types is challenging and a great variation between the methods’ performance was already highlighted.

The amplicon sequencing technique may lead to possible biases deriving from DNA extraction, PCR amplification, as well as the failure in discriminating between live or death cell. Furthermore, fungi produce different types of cells, such as simple cells, hyphae or spores, which may lead to biases during DNA extraction and either over- or underestimation. In addition, fungal sexual or asexual forms can be classified as different taxa with possible discrimination. The study of gut fungi is still a new science and remains a main challenge. Indeed, the metataxonomic approach is at presence a largely used method for characterizing the gut mycobiota. However, given the nature of PCR, bias can be introduced based on the target region chosen for the amplifications. Due to the observational design of this study, the presence of unmeasured confounding factors cannot be excluded. Reliable data on dietary habits of normoglycemic women were not available. The fecal samples were used as proxies for the microbiome of the entire gastrointestinal tract. The limitations of the food questionnaires must be recognized, even if the dietary intakes of our patients resembled those previously reported in pregnancy^75^. Finally, to the best of our knowledge, no previous studies have investigated the composition of the mycobiota neither in pregnancy nor in patients with GDM.

## Conclusions

Patients with GDM showed a predominance of fungal taxa with potential inflammatory effects, with a marked shift in their mycobiota during pregnancy, and complex bacteria-fungi interactions. If these data will be confirmed by further studies, the possibility to modulate the gut microbiome during pregnancy should be tested as an intriguing challenge to impact on the health of the mother and the offspring.

## Methods

### Participant enrolment

The first 50 patients consecutively diagnosed with GDM at the “Città della Salute e della Scienza” Hospital of Turin from April 2016, were enrolled. The characteristics of this cohort were already reported^30,76^. Briefly, inclusion criteria were: gestational age between 24 and 28 weeks, Caucasian race, GDM diagnosed by a 75 g oral glucose tolerance test (OGTT), performed in the morning, after at least 8 h-overnight fast, and interpreted according to international criteria^30^. The exclusion criteria were: twin pregnancy, use of prebiotics/probiotics, antibiotics or any drug during pregnancy, any pathological conditions before or during pregnancy (known diabetes mellitus, hypertension, cardiovascular, pulmonary, autoimmune, joint, liver or kidney diseases, thyroid dysfunction, cancer, any other disease/condition), no compliance to the study protocol.

A group of 150 normoglycemic women were then consecutively enrolled starting from October 2020, at the same Hospital. Inclusion criteria were: gestational age between 24 and 28 weeks, Caucasian race, normoglycemia diagnosed by a 75 g OGTT, according to the same criteria. Exclusion criteria were the same as for GDM patients.

### Ethical aspects

Each participant gave her written informed consent to participate in the study. The study protocol was approved by the Ethics Committee of the “Città della Salute e della Scienza” Hospital of Torino. All research was performed in accordance with the Helsinki Declaration principles.

### GDM patients

All GDM patients routinely received dietary counselling and nutritional and exercise recommendations in line with guidelines^77^. Questionnaires, anthropometric values, fasting blood samples and stool samples were collected for all participants both at 24–28 weeks of gestational age at the time of GDM diagnosis (second trimester, T2), and at 38 weeks, or before delivery, in the case of preterm delivery (third trimester, T3). Participants completed a 3-day food record (2 weekdays and 1 weekend day) at T2 and T3. Detailed information on how to record food and drink consumed by using common household measures was provided to all participants. Two dieticians checked all questionnaires for completeness, internal coherence, and plausibility.

Data relative to pre-pregnancy weight was self-reported; weight, height, and arterial blood pressure (BP) were measured at T2 and weight and BP at T3. Body weight was measured to the nearest 0.1 kg, and height was measured to the nearest 0.1 cm with a stadiometer (SECA model 711, Hamburg, Germany), with the participants wearing light clothes and no shoes. Arterial BP was measured from the left arm, in a sitting position, after at least 10 min of rest, with a mercury sphygmomanometer with appropriate cuff sizes (ERKA Perfect-Aneroid, Germany). Two measurements were taken by trained personnel with arm supported at heart level and the values reported were the means of the two.

A fasting blood sample was collected both at T2 and T3 from all the participants for the detection of serum glucose, glycated hemoglobin, insulin, total and HDL cholesterol, triglycerides, and C-reactive protein. Laboratory methods were previously reported^30^. All laboratory measurements were centralized. The HOMA-IR was calculated according to the published algorithm^78^.

### Normoglycemic women

Normoglycemic women were evaluated during the OGTT only (T2). Their weight, height, and arterial BP were measured as in the GDM patients.

### Stool samples

Stool samples were self-collected by the patients as previously described^30^. Briefly, the subjects were instructed to self-collect the samples, and all materials were provided in a convenient, refrigerated, specimen collection kit. Patients were provided with sterile containers to collect the feces (VWR, Milan, Italy). Fecal samples were collected at home and transferred into the sterile sampling containers using a polypropylene spoon (~ 10 g) and immediately stored at 4 °C. The specimens were transported to the laboratory within 8 h of collection under refrigerated temperature. Containers were immediately stored at − 80 °C for DNA extraction. No storage medium was used.

### DNA extraction and meta-taxonomic amplicon sequencing

Total DNA was extracted by using the RNeasy Power Microbiome KIT (Qiagen, Milan, Italy) following the manufacturer’s instructions. In order to digest the RNA, 5uL of RNAse-A was added to the mixture and then incubated for 1 h at 37 °C. DNA was quantified by using the QUBIT ds Kit and normalized at 5 ng/uL. 2.5 uL of the DNA was amplified by using the primers NL4R (5′-GGTCCGTGTTTCAAGACGG-3′) and LS2-MF (5′-GAGTCGAGTTGTTTGGGAAT-3′) as previously described^79^. Standard Illumina overhang adapter sequences were added to locus‐specific sequences. PCR consisted of 25 cycles (95 °C for 30 s, 55 °C for 30 s and 72 °C for 30 s) plus one additional cycle at 72 °C for 10 min as a final chain elongation. PCR products were then purified by using the Agencourt AMPure XP beads (Beckman Coulter Genomics) and tagged according to the Illumina Sequencing Library Preparation. Amplicons were quantified using Qubit dsDNA assay kit diluted to 4 nM, denatured with 0.2 N NaOH and spiked with 20% (v/v) of PhiX. The combination of pool library and PhiX were diluted to 8 pM, and paired end sequencing was performed on the MiSeq platform using the MiSeq Reagent Kit V2 (2 × 250 bp) (Illumina, San Diego, USA), following the standard Illumina sequencing protocol.

### Bioinformatics Analysis

After sequencing, fastq files were imported in QIIME version 2. Sequence adapters and primers were trimmed by using *cutadapter*, while DADA2 algorithm was used to trim low quality reads, to remove chimeric sequences, and joined sequences shorter than 300 bp by using the DADA2 denoise paired plug-in of QIIME2. Amplicon sequence variants (ASVs) obtained by DADA2 were used for taxonomic assignment using the QIIME feature-classifier plugin against the manually build database for the mycobiota^79^. Briefly the database was constructed using the large subunit rRNA gene sequences, 23.381 sequences were downloaded from Silva database and from NCBI. Taxonomy assignment for 26S was double checked on BLAST suite tools to confirm the taxonomic assignment.

### Statistical analyses

QIIME2 diversity script was used to perform alpha and beta diversity analysis. Bray–Curtis distance matrix was generated by QIIME2 and was used both to build the principal coordinate analysis (PCoA) and to perform pairwise PERMANOVA by the “vegan” package in R environment. Principal Component Analysis (PCA) based on ASVs at genus level on GDM dataset was performed and plotted through the function *dudi.pca* in R environment. Not-normally distributed variables were presented as median (range interquartile). Within-differences in GDM patients (T2 *vs* T3) were evaluated by paired-sample t-test, or Wilcoxon matched pairs test, as appropriate. Differences between categorical variables were computed by the chi-square test. Between-differences between GDM patients and normoglycemic women were assessed by t-Student’s test or Mann–Whitney test.

q2-sample-classifier plugin of QIIME2 was used to predict sample type (GDM/normoglycemic) as a function of the amplicon sequence variants detected in the samples. Briefly, the input samples were randomly split into a training set and a test set. The test set was held out until the end of the pipeline, allowing to assess accuracy. The model was trained to predict a specific target variable (GDM vs normoglycemic women) based on the Random Forest approach. The accuracy of the results was calculated from the qiime2 software using the Receiver Operating Characteristic (ROC) curves as well as by measure the area under the curve (AUC) in order to verify the classification accuracy of the model.

Pairwise Spearman’s non-parametric correlations were used to study the relationships between the relative frequency of fungal taxa, bacteria, and dietary and clinical variables. Bacteria composition of GDM cohort previously published^30^ was used to performed the correlation analysis. Correlation network was visualized by using Cytoscape v. 3.8.2. P-values were adjusted for multiple testing using the Benjamini–Hochberg procedure, which assesses the false discovery rate (FDR). Multiple regression analyses were performed to evaluate the associations between fungal relative frequency and nutrient and clinical variables in GDM patients, after adjusting for age and BMI. The same model was used to evaluate the association of specific taxa with GDM in the whole sample of women (Statistica, ver. 7.0; StatSoft Inc., Tulsa, OK, USA).

## Supplementary Information

Supplementary Information.

## Data Availability

Data generated by sequencing were deposited in the National Center for Biotechnology Information (NCBI) Sequence Read Archive (SRA) and are available under the BioProject Accession Number PRJNA762265 (https://www.ncbi.nlm.nih.gov/bioproject/PRJNA762265).
