# Supplementary material for: Mycobiota composition and changes across pregnancy in patients with gestational diabetes mellitus (GDM)
Source: Sci Rep. 2022 Jun 2;12:9192. doi: 10.1038/s41598-022-13438-0 (PMC9163055; doi:10.1038/s41598-022-13438-0)
Supplement: Supplementary file 1 — Supplementary Information. [file 41598_2022_13438_MOESM1_ESM.docx]

## Mycobiota composition and changes across pregnancy in patients with gestational diabetes mellitus (GDM)

## Ilario Ferrocino*^1^. Valentina Ponzo^2^. Marianna Pellegrini^2^. Ilaria Goitre^1^. Matteo Papurello^2^. Irene Franciosa^1^. Chiara D’Eusebio^2^. Ezio Ghigo^2^. Luca Cocolin^1^. Simona Bo*^2^

## 1 Department of Agricultural. Forestry and Food Science. University of Torino. 10095 Grugliasco. Torino. Italy;

## 2 Department of Medical Sciences. University of Torino. 10126 Torino. Italy;

## * Correspondence: [ilario.ferrocino@unito.it](mailto:ilario.ferrocino@unito.it) (I.F.); [simona.bo@unito.it](mailto:simona.bo@unito.it) (S.B.)


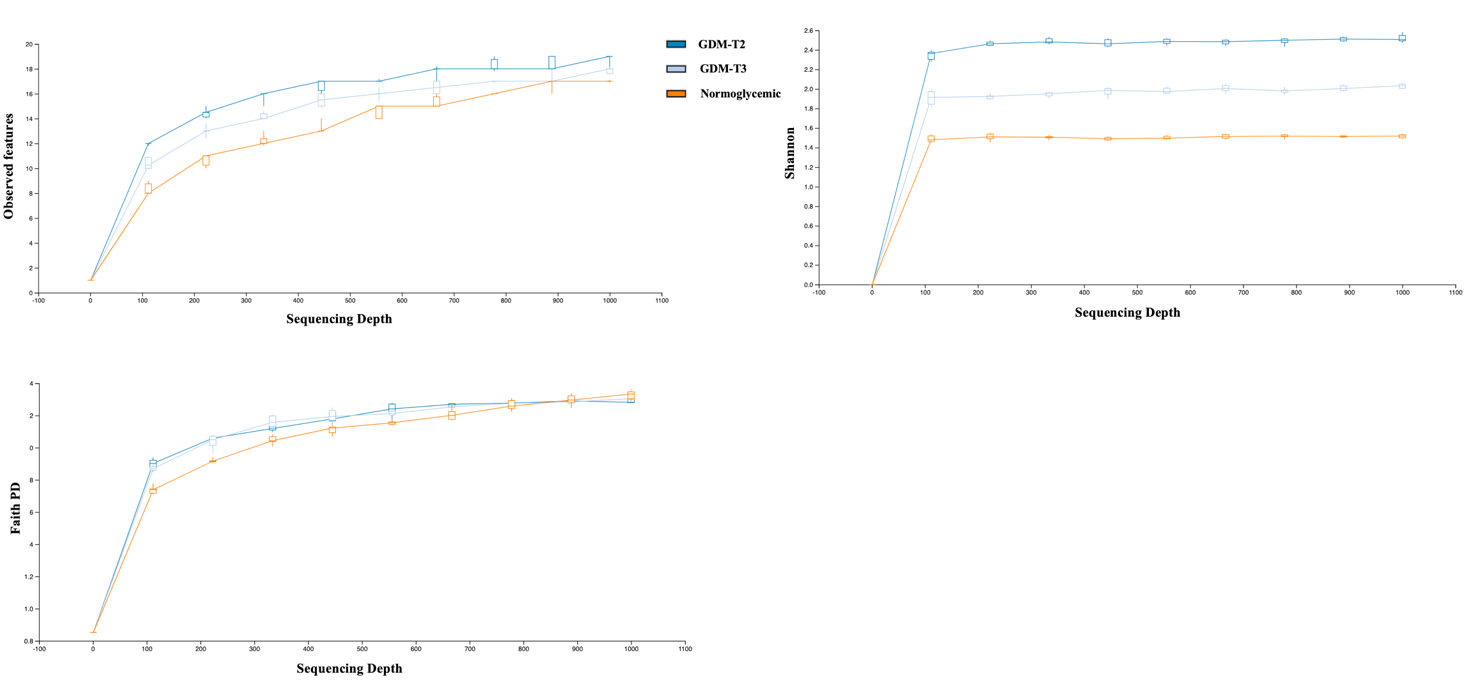


**Fig. S1**. Rarefaction curves of fungal sequence coverage in normoglycemic and GDM women during the second and third trimester of pregnancy. The curve depicts the number of observed features, the Shannon and Faith PD index (y-axis) and the number of sequences (x-axis).


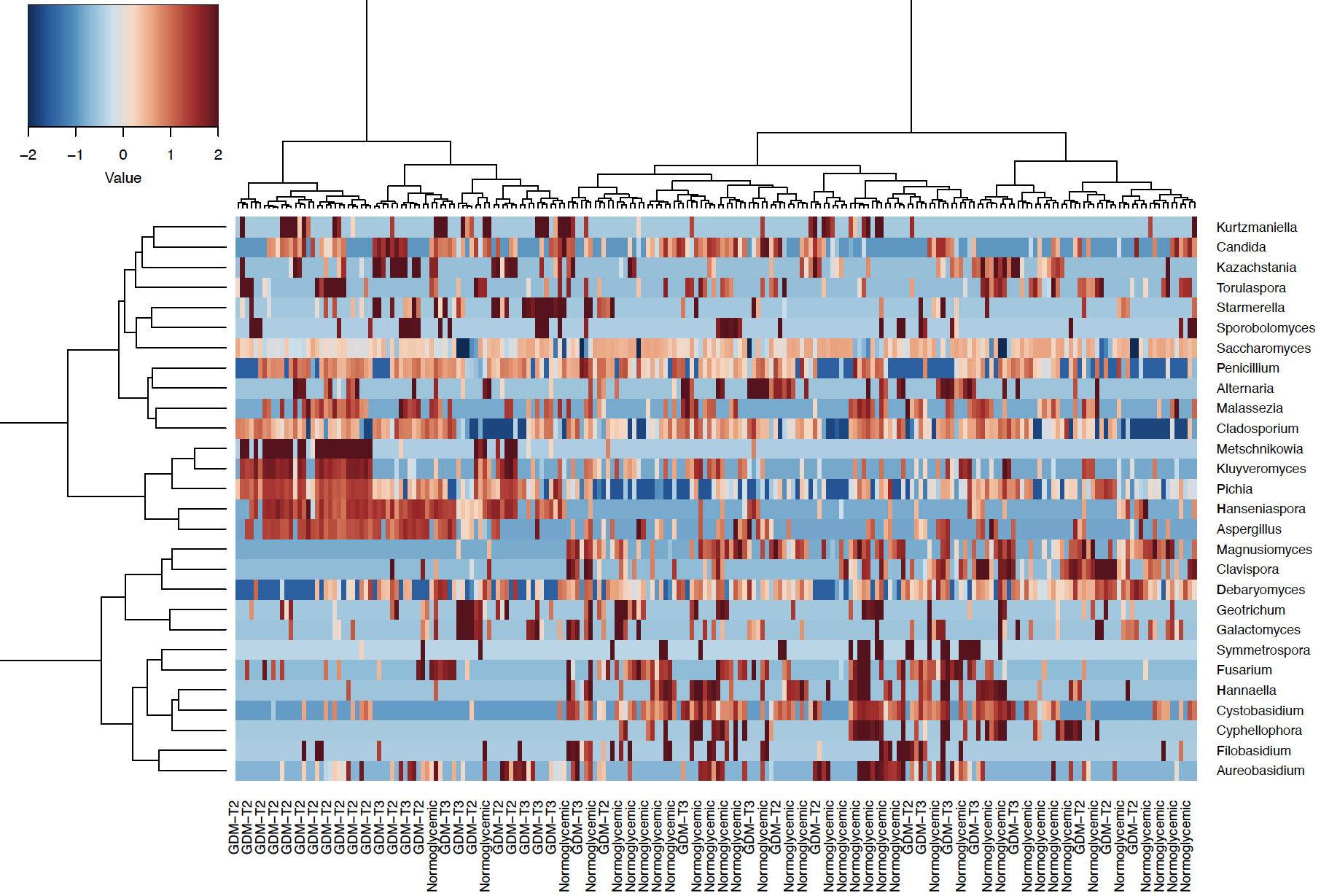


**Fig. S2**. Heatmap of gut mycobiota of normoglycemic and GDM women during the second and third trimester of pregnancy. Only ASVs genera with a relative abundance >1% in at least 10% of subjects are shown. Rows and columns are clustered by Ward linkage hierarchical clustering.

**Supplementary Table 1.** Characteristics of the participants

|  | **GDM** | | **Normoglycemic** |
| --- | --- | --- | --- |
|  | **T2** | **T3** | **T2** |
| Number | 41 | 41 | 121 |
| Age | 37.1±4.2 |  | 35.2±4.3** |
| Pre-pregnancy weight (kg) | 69.3±14.6 |  | 60.7±9.2** |
| Pre-pregnancy BMI (kg/m^2^) | 25.8±5.9 |  | 22.4±3.2** |
| Nulliparous (%) | 58.5 |  | 53.7 |
| Weight (kg) | 75.8±12.9 | 79.0±13.3* | 67.3±9.4** |
| BMI (kg/m^2^) | 28.2±5.3 | 29.4±5.4* | 24.8±3.2** |
| Systolic BP (mmHg) | 110.8±11.7 | 116.1±11.6* | 110.1±11.6 |
| Diastolic BP (mmHg) | 72.9±7.5 | 75.8±9.1 | 71.5±6.9 |
| Fasting glucose (mg/dL) | 97.9±19.2 | 96.6±19.1 | 78.6±5.3** |
| HbA1c (%) | 4.6±0.8 | 4.9±0.8 |  |
| Fasting insulin (µU/mL) | 10.1 (8.4) | 11.6 (10.0)* |  |
| HOMA-IR (mmol/L*µU/mL) | 2.3 (1.9) | 2.8 (2.7) |  |
| Total cholesterol (mg/dL) | 234.1±32.4 | 257.0±48.6* |  |
| HDL-cholesterol (mg/dL) | 65.8±13.4 | 67.0±15.6 |  |
| Triglycerides (mg/dL) | 173.3±53.0 | 259.2±70.5* |  |
| CRP (mg/L) | 4.1 (4.2) | 4.5 (7.5)* |  |
| ***Dietary intakes*** |  |  |  |
| Energy (kcal) | 1605.8±254.4 | 1766.1±306.7* |  |
| Carbohydrates (% total kcal) | 44.4±6.6 | 43.1±6.4 |  |
| Sugars (% total kcal) | 8.8±4.7 | 6.2±4.5* |  |
| Sugars (g/day) | 35.3±20.1 | 27.9±21.3 |  |
| Oligosaccharides (g/day) | 36.7±19.7 | 54.2±23.2* |  |
| Starch (g/day) | 107.3±28.9 | 109.7±38.7 |  |
| Fiber (g/day) | 14.5±4.2 | 15.1±5.3 |  |
| Proteins (% total kcal) | 15.6±2.3 | 16.6±5.3 |  |
| Total fats (% total kcal) | 42.2±5.2 | 42.3±6.3 |  |
| SFA (% total kcal) | 11.3±2.2 | 11.1±2.7 |  |
| PUFA (%kcal) | 4.9±1.7 | 4.4±1.1 |  |

BMI=body mass index. BP=blood pressure. HbA1c= glycated hemoglobin. HOMA-IR=Homeostasis Model Assessment-Insulin Resistance. HDL=high density lipoprotein. LDL=low-density lipoprotein. CRP=C-reactive protein. SFA=saturated fatty acids. PUFA=polyunsaturated fatty acids. Values are expressed as mean ± standard deviation or median (interquartile range); *p<0.05 within-GDM groups (T2 *vs* T3); **p<0.05 between GDM (T2) *vs* normoglycemic women.

**Supplementary Table 2.** Relative frequency of Amplicon Sequence Variants (ASVs).

| **ASVs** | **GDM** | | **Normoglycemic** |  |
| --- | --- | --- | --- | --- |
|  | **T2** | **T3** | **T2** |  |
| *Alternaria* | 0.239±0.648 | 0.793±2.439 | 0.664±2.322 |  |
| *Aspergillus* | 0.960±1.027 | 1.376±2.441 | 1.750±8.789 |  |
| *Aureobasidium* | 0.643±2.001 | 0.251±0.636 | 0.564±1.447 |  |
| *Candida* | 2.564±7.589 | 5.722±15.525 | 1.969±6.660 |  |
| *Cladosporium* | 2.337±4.139 | 5.335±7.442 | 3.242±6.126 |  |
| *Clavispora* | 0.417±2.752 | 0.123±0.608 | 0.628±1.722 |  |
| *Cyphellophora* | 0.004±0.025 | 0.010±0.068 | 0.815±2.482 |  |
| *Cystobasidium* | 0.279±0.918 | 1.034±4.175 | 2.117±3.948 |  |
| *Debaryomyces* | 4.236±12.144 | 2.669±7.034 | 3.857±8.150 |  |
| *Filobasidium* | 0.178±0.771 | 0.003±0.013 | 0.305±1.171 |  |
| *Fusarium* | 0.393±1.638 | 0.450±1.332 | 0.748±2.131 |  |
| *Galactomyces* | 0.033±0.111 | 3.244±13.236 | 1.262±5.956 |  |
| *Geotrichum* | 0.096±0.342 | 2.911±12.380 | 0.928±5.372 |  |
| *Hannaella* | 0.051±0.335 | 0.070±0.380 | 0.787±2.551 |  |
| *Hanseniaspora* | 1.743±1.891 | 1.630±2.845 | 0.657±5.958 |  |
| *Kazachstania* | 0.135±0.368 | 0.492±0.969 | 0.260±1.252 |  |
| *Kluyveromyces* | 3.188±3.160 | 0.057±0.184 | 0.445±2.031 |  |
| *Kurtzmaniella* | 0.422±1.545 | 0.445±1.062 | 0.101±0.375 |  |
| *Magnusiomyces* | 0.784±5.237 | 0.461±3.059 | 1.302±4.224 |  |
| *Malassezia* | 0.322±0.580 | 0.701±1.613 | 0.666±3.691 |  |
| *Metschnikowia* | 3.692±5.779 | 0.009±0.057 | 0.000±0.000 |  |
| *Penicillium* | 2.309±2.665 | 5.756±15.638 | 3.082±9.048 |  |
| *Pichia* | 16.125±12.414 | 3.791±12.993 | 1.908±6.131 |  |
| *Saccharomyces* | 54.274±23.303 | 57.012±27.810 | 68.098±31.017 |  |
| *Sporobolomyces* | 0.054±0.209 | 0.412±1.027 | 0.102±0.528 |  |
| *Starmerella* | 0.023±0.079 | 1.306±3.106 | 0.065±0.269 |  |
| *Symmetrospora* | 0.208±0.822 | 0.030±0.168 | 0.381±1.992 |  |
| *Torulaspora* | 2.227±4.530 | 1.134±7.529 | 0.384±2.687 |  |

Mean ± standard deviation of relative frequency (%) detected by amplicon target sequencing in normoglycemic and GDM women during the second and third trimester of pregnancy. Only ASVs genera with a relative frequency >1% in at least 10% of subjects are shown.
